# Hyperexcitability and translational phenotypes in a preclinical mouse model of *SYNGAP1-*related intellectual disability

**DOI:** 10.1038/s41398-024-03077-6

**Published:** 2024-10-02

**Authors:** Timothy A. Fenton, Olivia Y. Haouchine, Elizabeth B. Hallam, Emily M. Smith, Kiya C. Jackson, Darlene Rahbarian, Cesar P. Canales, Anna Adhikari, Alex S. Nord, Roy Ben-Shalom, Jill L. Silverman

**Affiliations:** 1 MIND Institute, University of California Davis School of Medicine, Sacramento, CA 95817 USA; 2 Department of Psychiatry and Behavioral Sciences, University of California Davis School of Medicine, Sacramento, CA 95817 USA; 3 UC Davis Center for Neuroscience; Department of Psychiatry and Behavioral Sciences & Department of Neurobiology, Physiology and Behavior, University of California Davis, Davis, CA 95616 USA; 4 Department of Neurology, University of California Davis School of Medicine, Sacramento, CA 95817 USA

**Keywords:** Neuroscience, Prognostic markers, Autism spectrum disorders

## Abstract

Disruption of *SYNGAP1* directly causes a genetically identifiable neurodevelopmental disorder (NDD) called SYNGAP1-related intellectual disability (SRID). Without functional SynGAP1 protein, individuals are developmentally delayed and have prominent features of intellectual disability (ID), motor impairments, and epilepsy. Over the past two decades, there have been numerous discoveries indicating the critical role of *Syngap1*. Several rodent models with a loss of *Syngap1* have been engineered, identifying precise roles in neuronal structure and function, as well as key biochemical pathways key for synapse integrity. Homozygous loss of *SYNGAP1/Syngap1* is lethal. Heterozygous mutations of *Syngap1* result in a broad range of behavioral phenotypes. Our in vivo functional data, using the original mouse model from the Huganir laboratory, corroborated behaviors including robust hyperactivity and deficits in learning and memory in young adults. Furthermore, we described impairments in the domain of sleep, characterized using neurophysiological data that was collected with wireless, telemetric electroencephalography (EEG). *Syngap1*^+/−^ mice exhibited elevated spiking events and spike trains, in addition to elevated power, most notably in the delta power frequency. For the first time, we illustrated that primary neurons from *Syngap1*^+/−^ mice displayed: 1) increased network firing activity, 2) greater bursts, 3) and shorter inter-burst intervals between peaks, by utilizing high density microelectrode arrays (HD-MEA). Our work bridges in vitro electrophysiological neuronal activity and function with in vivo neurophysiological brain activity and function. These data elucidate quantitative, translational biomarkers in vivo and in vitro that can be utilized for the development and efficacy assessment of targeted treatments for SRID.

## Introduction

Many severe neurodevelopmental disorders (NDDs) include underlying excitatory/inhibitory imbalances and seizures. These underlying imbalances are thought to worsen behavioral symptoms of NDDs, such as autism spectrum disorders (ASD) and intellectual disabilities (ID) and underlie cognitive decline and impaired cognitive development. The *SYNGAP1* (synaptic Ras GTPase activating protein) gene encodes the protein (SynGAP1), which is selectively expressed in the brain, and highly enriched at excitatory synapses [1, 2], and is critical for neuronal development and synaptic plasticity [3].

Detailed research on SynGAP1 since its description in 1998 has produced high quality data on its own protein structure, role in neuronal structure, biochemical and physiological function, and its unique neuronal localization [4, 5]. Reduction or loss of SynGAP1 leads to Ras activation and excessive AMPA receptor incorporation into the cell membrane [6], components critical for long‐term potentiation, dendritic spine formation, neuronal development and structural integrity, neuronal signaling, synaptic strength or dysregulation [4], and the long term potentiation processes that underlie cognition and excitability [5].

SYNGAP1-related intellectual disability (SRID) is an NDD characterized by global developmental delay, ASD, ID, and epilepsy. In most individuals, the ID is moderate to severe, and the epilepsy is either generalized or has myoclonic absence events. Loss of its function at the synapse results in dysfunctional and aberrant neuronal signaling. SRID can therefore be considered a ‘synaptopathy’, which refers to disorders caused by synaptic dysfunction that leads to aberrant neuronal communication [7]. In addition to the dysregulation of synaptic signaling, loss of SynGAP1 also results in aberrant Ras GTPase cellular signaling, making SRID, a Rasopathy. The ‘Rasopathies’ refer to a group of brain disorders in which the RAS/MAPK signaling pathway is disrupted. This dual dysregulation of inter- and intracellular communication results in debilitating and severe consequences clinically. De novo loss of *SYNGAP1* have been found in patients with developmental delays and ID (96%), epilepsy (98%), and/or ASD (50%) [8, 9].

As SynGAP1 is a negative regulator of excitatory neurotransmission, overexpression of SynGAP1 results in a dramatic loss of synaptic efficacy. Conversely, enhanced synaptic transmission occurs when SynGAP1 is disrupted by RNA interference [4], highlighting the fact that SynGAP1 is critical for multiple processes, and has an essential role at the synapse and in cellular signaling. As described by Creson and Rumbaugh et al. [5, 10], SynGAP1 is modifiable with a variety of modern technologies, so restoration of SynGAP1 is not a hopeless endeavor. Targeted molecular strategies and therapeutics, including adeno associated viral vectors [9], CRISPRa activation [11], activating RNAs [12], and antisense oligonucleotides (ASOs) [13] are in development. In addition to precision therapeutics, SRID, being adjacent to Rasopathies and Synaptopathies, widens opportunities to repurpose traditional pharmacologic compounds.Given the outstanding need to develop effective therapies for SRID, our laboratory has been focused on biomarkers and functional outcome measures that are rigorous, robust, reliable, and objective. Critically, herein, we provide a report of reproduced and extended behavioral impairments resulting from the loss of *Syngap1*, reduced time in slow wave sleep and elevated time in the active wake sleep stage, in addition to elevated delta power spectral density. These data confirm a unique electrophysiological signature in live mice, missing a copy of *Syngap1*, as well as in cultured primary cortical neurons from these mice, bridging electrophysiological single neuronal network firing patterns in vitro to neurophysiological and behavioral phenotypes in vivo. The HD-MEA work reported here is the first report of these electrophysiological properties on a network scale in *Syngap1* deficient neurons. These data pave the way for cellular biomarkers to potentially bridge the gap between mouse primary neurons and human neural progenitor stem cells, reprogrammed from human iPSCs.

## Methods

### Animals

All animals were housed in Techniplast cages (Techniplast, West Chester, PA, USA) in a temperature (68–72 °F) and humidity ( ~ 25%) controlled vivarium maintained on a 12:12 light-dark cycle. All procedures were approved by the Institutional Animal Care and Use Committee (IACUC) at the University of California Davis and were conducted in accordance with the National Institutes of Health Guide for the Care and Use of Laboratory Animals. B6;129-*Syngap1*^tm1Rlh^/J mice were obtained from The Jackson Laboratory (JAX #008890, Bar Harbor, ME, USA) and fed a standard diet of Teklad global 18% protein rodent diets 2918 (Envigo, Hayward, CA, USA). Rodent chow and tap water were available *ad libitum*. In addition to standard bedding, a Nestlet square, shredded brown paper, and a cardboard tube (Jonesville Corporation, Jonesville, MI) were provided in each cage. Heterozygous deletion male mice B6;129-Syngap1tm1Rlh/J (Jax Mice # 008890) (*Syngap1*^+/−^) were bred with hybrid females (WT, *Syngap1*^+/+^) to generate mutant (*Syngap1*^+/−^) and wildtype 129S1-C57BL/6 J F1 (WT, *Syngap1*^+/+^) littermates. The B6;129-Syngap1 hybrid mice are the offspring of a cross between C57BL/6 J (JAX #006644) females and 129S1/SvImJ males (JAX #101043) and were utilized to increase viability of *Syngap1* heterozygotes and reduce strain-based biases such as seizure resistance/vulnerability. To identify mice, pups were labeled by paw tattoo on postnatal days (PND) 2–4 using non-toxic animal tattoo ink (Ketchum Manufacturing Inc., Brockville, ON, Canada). Tails of pups were clipped (1–2 mm) for genotyping following the UC Davis IACUC policy regarding tissue collection. Genotyping was performed with REDExtract-N-Amp (Sigma Aldrich, St. Louis, MO, USA) using primers from the Jackson Laboratory oIMR9462 ATGCTCCAGACTGCCTTGGGAAAAG, oIMR9463 ACCTCAAATCCACACTCCTCTCCAG, and oIMR9464 AGGGAACATAAGTCTTGGCTCTGTC.

To reduce carry-over effects from repeated behavioral testing, at least 24 h were allowed to pass between the completion of one task and the start of another. Assays were performed in the order of least to most stressful and between the hours of 8:00 AM PST and 7:00 PM PST during the light phase. All behavior testing was conducted by an experimenter blinded to genotype and included both sexes. Mice were allowed to habituate in their home cages in a dimly lit room adjacent to the testing room for 1 h prior to the start of testing to limit any effect of transporting between the vivarium and testing suite. Between subjects, testing apparatus surfaces were cleaned using 70% ethanol and allowed to dry. To achieve adequate animal numbers for the behavioral cohort, 13 litters were used, to obtain a powered sample size *N* = 12 WT males, *N* = 12 WT females, *Syngap1*^+/−^ *N* = 12 males, and *Syngap1*^+/−^ *N* = 12 females. Behavioral testing began when mice were 8 weeks of age (postnatal day (PND) 55). The order of testing was (1) elevated plus-maze, (2) light ↔ dark transitions test, (3) open field, (4) spontaneous alternation, and (5) novel object recognition.

### Protein extraction and western blot analysis

Brains were extracted from PND42 mice. A total of 6 mouse brains across 2 litters were used to obtain the samples. The cortex was collected then snap-frozen on dry ice for storage at -80°C. The cortical tissue was suspended in 600 μl lysis buffer (50 mM Tris-HCl, 140 mM NaCl, 10% Glycerol, 0.5% IGEPAL, 0.25% Triton X-100, protease inhibitor cocktail (Roche, 4693124001)) and manually homogenized. After a 30 min incubation on ice, samples were lysed with a probe sonicator (Qsonica CL-18, 20% amplitude, 10 cycles of 5 s on/off intervals) and placed back on ice to incubate for another 30 min. Cell debris was collected by centrifugation (14,000 g, 4°C, 10 min) and the supernatant was moved to a new tube. Protein concentration was quantified using a BCA protein assay kit (Pierce, 23225) and samples were stored at −80 °C until use.

Protein lysates were diluted in 30 μl 6X Laemmli SDS buffer (375 mm Tris-HCl, 9% SDS, 50% glycerol, 0.03% Bromophenol blue) and 5% β-mercaptoethanol, boiled at 70°C for 10 minutes, and separated on 4–20% polyacrylamide tris-glycine protein gel (BioRad, 4561094). The separated proteins were transferred onto a PVDF membrane (Millipore Sigma, 0.45μm, IPFL00010) by wet transfer (overnight, constant 13 mA at max voltage, 4 °C). Membranes were blocked with Intercept phosphate buffered saline (PBS) blocking buffer (Li-Cor, LIC-927-90001) at room temperature for one hour. We used a previously validated antibody [12, 14] for total SynGAP1 (Invitrogen, PA1-046) and normalized to a validated GAPDH antibody (Sigma-Aldrich G8795). Primary antibodies were diluted in 7.5 ml Intercept PBS blocking buffer with 0.1% Tween (SynGAP1; 1:1000dil, Gapdh; 1:15000). Membranes were incubated with the primary antibody solution overnight at 4 °C, then washed four times for 5 min with PBS with 0.1% Tween (PBST). Fluorescently tagged secondary antibodies (Li-Cor, 926-32212 and 926-68023) were diluted in 10 ml Intercept PBS blocking buffer with 0.1% Tween. After the initial washes, blots were incubated with the secondary antibody solution for one hour at room temperature. Blots were washed an additional four times for 5 min with PBST and two times with PBS. Bands were visualized using the Odyssey DLx imaging system (Li-Cor). Acquired images were processed in ImageJ (Java 1.8.0_172) to quantify intensity of the predominant SynGAP1 band and the Gapdh band. SynGAP1 protein expression was normalized to Gapdh protein expression by dividing the SynGAP1 band intensity by the Gapdh band intensity from the same sample. The average SynGAP1 protein expression from *Syngap1*^+/+^ mice was then set to one to quantify the percentage difference of protein expression between *Syngap1*^+/+^ and *Syngap1*^+/−^ mice.

### Elevated-plus maze

The elevated-plus maze (EPM) is a well-established task for assessing anxiety-like conflict behavior in rodents by allowing mice to choose between entering the two open arms of the maze (natural exploratory drive) or entering and remaining in the safety of the two closed arms. All four arms are elevated 1 m from the floor, with the drop-off detectable only in the open arms. The EPM was performed according to previously described procedures [15–18] using a mouse EPM (model ENV-560A) purchased from Med Associates (St. Albans, VT). The EPM contained two open arms (35.5 cm × 6 cm) and two closed arms (35.5 cm × 6 cm) radiating from a central area (6 cm × 6 cm). A 0.5 cm high lip surrounded the edges of the open arms, whereas the closed arms were surrounded by 20 cm high walls. The EPM was cleaned with 70% ethanol before the beginning of the first test session and after each subject mouse was tested, with sufficient time for the ethanol to dry and for the odor to dissipate before the start of the next test session. The room was illuminated at ~ 40 lx. To begin the test, the mouse was placed in the central area facing the open arm. The mouse was allowed to freely explore for 5 min during which time the activity was recorded by a computer counting beam breaks between arms. Subjects from 13 litters completed this task, with a final sample size per group of, *N* = 12 WT males, *N* = 12 WT females, *Syngap1*^+/−^ *N* = 12 males, and *Syngap1*^+/−^ *N* = 12 females. Genotype differences were analyzed using unpaired Student's t-tests.

### Light ↔ dark transitions

The light ↔ dark transitions test assesses anxiety-like conflict behavior in mice by evaluating the tendency of mice to avoid brightly lit areas versus their strong tendency to explore a novel environment. The light ↔ dark transitions test was performed in accordance with previously described procedures [15–18]. The test began by placing the mouse in the light side ( ~ 320 lx; 28 cm × 27.5 cm × 27 cm) of an automated 2-chambered apparatus, in which the enclosed/dark side ( ~ 5 lx; 28 cm × 27.5 cm × 19 cm) was reached by traversing the small opening of the partition between the two chambers. The mouse was allowed to explore freely for 10 min. Time in the dark side chamber and total number of transitions between the light and dark side chambers were automatically recorded during the 10 min session using Labview 8.5.1 software (National Instruments, Austin, TX). Subjects from 13 litters completed this task. Outliers were removed following a Grubb’s outlier analysis to create the final sample size per group of *N* = 10 WT males, *N* = 9 females, *Syngap1*^+/−^ *N* = 12 males, and *Syngap1*^+/−^ *N* = 10 females. Genotype differences were analyzed between genotypes using an unpaired Student’s t-test.

### Open field

General exploratory locomotion in a novel open field environment was assayed in an arena sized 40 cm × 40 cm × 30.5 cm, as previously described [15–17, 19–24]. Open field activity was considered an essential control for effects on physical activity, for example, as sedation or hyperactivity could confound the interpretation of interaction time with an arena or objects. The testing room was illuminated at ~40 lx. Subjects from 13 litters completed this task, with a final sample size per group of, *N* = 12 WT males, *N* = 12 WT females, *Syngap1*^+/−^ *N* = 12 males, and *Syngap1*^+/−^ *N* = 12 females. Horizontal activity, total activity, vertical activity, and center time was measured and analyzed with a two-way repeated measures ANOVA, and subsequent comparison via Sidak’s post hoc test between genotypes. When activity across the 30 min session was summed, a Student’s unpaired t-test was performed between genotypes.

### Spontaneous alternation

Spontaneous alternation in a Y-maze was assayed using methods modified from previous studies [15, 16, 19]. The Y-maze assesses working memory and learning [25–28] in which subjects explored a Y-shaped maze constructed of matte white acrylic (P95 White, Tap Plastics, Sacramento, CA, USA) for 8 minutes and were recorded from an overhead camera with the behavioral tracking software Ethovision XT. Mice were placed at the center of the initial arm facing the center of the maze. Percentage of spontaneous alternations is calculated as the number of triads (entry into three different arms without returning to a previously entered arm) relative to the number of alternation opportunities. Subjects from 13 litters completed this task, with a final sample size per group of, *N* = 12 WT males, *N* = 12 WT females, *Syngap1*^+/−^ *N* = 12 males, and *Syngap1*^+/−^ *N* = 12 females. Genotype differences were analyzed between genotypes using an unpaired Student’s t-test. All scoring was conducted by an observer blinded to genotype.

### Novel object recognition

The novel object recognition (NOR) test was conducted in opaque matte white (P95 White, Tap Plastics, Sacramento, CA) open field arenas (41 cm × 41 cm × 30 cm), using methods similar to those previously described [15, 17]. The experiment consisted of 4 sessions: a 30 min exposure to the open field arena the day before the test, a 10 min re-habituation on test day, a 10 min familiarization session and a 5 min recognition test. On day 1, each subject was habituated to a clean, empty open field arena for 30 min. 24 h later, each subject was returned to the open field arena for another 10 min for the habituation phase. The mouse was then removed from the open field and placed in a clean temporary holding cage for approximately 2 min. Two identical objects were placed in the arena. Each subject was returned to the open field in which it had been habituated and allowed to freely explore for 10 min. After the familiarization session, subjects were returned to their holding cages, which were transferred from the testing room to a nearby holding area. The open field was cleaned with 70% ethanol and let dry. One clean familiar object and one clean novel object were placed in the arena, where the two identical objects had been located during the familiarization phase. 60 min after the end of the familiarization session, each subject was returned to its open field for a 5 min recognition test, during which time it was allowed to freely explore the familiar object and the novel object. The familiarization session and the recognition test were recorded with Ethovision XT video tracking software and manually scored by an experimenter blinded to genotype (Version 9.0, Noldus Information Technologies, Leesburg, VA). Object investigation was defined as time spent sniffing the object when the nose was oriented toward the object and the nose–object distance was 2-cm or less. Recognition memory was defined as spending significantly more time investigating the novel object compared to the familiar object using within genotype paired Student’s t-test. Total time spent sniffing both objects was used as a measure of general exploration. Time spent sniffing two identical objects during the familiarization phase confirmed the lack of an innate side bias. Objects used were plastic toys: a small soft plastic orange safety cone and a hard plastic magnetic cone with ribbed sides, as previously described [15, 16, 19, 29, 30]. Subjects from 13 litters completed this task, with a final sample size per group of, *N* = 12 WT males, *N* = 12 WT females, *Syngap1*^+/−^ *N* = 12 males, and *Syngap1*^+/−^ *N* = 12 females.

### Electroencephalography (EEG)

To capture electroencephalography data, an independent cohort of 19 male mice (*N* = 9 WT and *N* = 11 SynGap1^-/+^) were surgically implanted with wireless EEG telemetric devices (HD-X02, Data Sciences International, St. Paul, MN, USA), as previously described [15, 20, 21, 31]. The implantation procedure was performed in accordance with the UC Davis IACUC Guidelines for Rodent Survival Surgery. All mice aged 2–4 months old and weighing over 20 g, were anesthetized with vaporized liquid isoflurane (Piramal Critical Care, Inc., Bethlehem, PA, USA).

With a micro drill (Stoelting), two 1 mm-diameter burr holes were manually drilled (1.0 mm anterior and 1.0 mm lateral; −3.0 mm posterior and 1.0 mm lateral relative to bregma) allowing for the placement of two steel surgical screws (00–96 × 1/16 IROX screw, DSI, MN, USA). A subcutaneous pocket lateral to the spine was then made using a Crile Hemostat, minimizing excess tissue damage, and avoiding potential discomfort to the animal once implanted. Attached to the implant were two pairs of reference and sensing leads made of a nickel cobalt-based alloy insulated with medical-grade silicone, used to collect EEG and EMG biopotential data. One set of leads, used to measure EEG activity across the frontal cortical area, were individually attached to a surgical screw by removing the silicone insulation from the terminal end of the lead and tying the exposed wire around the base of the screw. The remaining set of leads were used to measure EMG activity. Animals were housed in a temperature controlled ventilated cabinet (Aria Bio-C36 EVO Ventilated Cabinet, Techniplast, Maggio, Italy) to support the maintenance of core body temperature by limiting activity dependent thermoregulation to improve postoperative recovery. Analgesic (carprofen) was administered the day after surgery, and as necessary following a thorough health evaluation performed twice a day during the 7–10-day postoperative period.

Untethered EEG activity was recorded in the home cage of individually housed, freely moving mice each assigned to a PhysioTelTM RPC-1 receiver plate (DSI, MN, USA). EEG and EMG data were collected at a sampling rate of 500 Hz with a 0.1 Hz high-pass and 100 Hz low-pass bandpass filter. The signal was transmitted to a control box which facilitates the transformation of signal between each implant and receiver pairing to the acquisition computer running Ponemah software (DSI, MN, USA). Activity, temperature, and signal strength were collected at a sampling rate of 200 Hz.

Acquisition of .edf files from DSI’s Ponemah™ were loaded into DSI’s analysis software titled Neuroscore™ for conversion of the signal to a numerical output. A Fast Fourier Transformation, 30 s epochs, and Hamming window 0 Hz–50 Hz were used. Spiking was defined by an absolute threshold of 200 µV, a minimum spike duration of 1 ms and a maximum spike duration of 200 ms. The spike interval minimum was 0.05 s while the spike interval maximum was 0.5 s, the minimum number of spikes was 3 and spiking time equal to 1 s to define a spike train. Our low threshold avoids floor effect. For power spectral density (PSD), frequency increments at 0.05 Hz early and then binned into 2 Hz segments once a steady signal was secured. For individual power bands, the entirety of band throughout the duration of recording was averaged, using 10 s epochs.

The sleep data utilizes the same signal acquired by EEG as described above. The data spans 72 h. For sleep analysis, our detection levels for delta power used a maximum probability of paradoxical sleep/wake at a delta ratio of 0.5 and maximum probability of slow wave sleep at delta ratio of 1. Theta power used maximum probability wake/slow wave sleep at a theta to delta ratio of 1.3 and maximum probability of paradoxical sleep at theta to delta ratio of 3. EMG power played a substantial role in designating sleep stages. EMG used a maximum probability of paradoxical sleep/slow wave sleep at an EMG ratio 1:1 and a maximum probability of wake at EMG ratio of 2.4. All activity levels above 0.1 scored were scored as “Active Wake”, the score when the EMG power level is 1.5 times the maximum set EMG probability level. Signal was considered artifact at 0.2 mV EEG and 1 mV EMG. Delta, Theta, EMG, and accelerometer data all equally contribute to sleep stage analysis.

### Cell culture

Primary cortical neuron-glia cultures were prepared using brain tissue from PND0-1 *Syngap1*^+/−^ pups as previously described [32, 33]. Briefly, cortices were minced with a razor blade prior to incubation in Hibernate A containing papain and DNase. The tissue was then triturated with glass pipettes to dissociate cells from the extracellular matrix (Bellco Glass, Vineland, NJ). Dissociated cells were counted and plated on microelectrode array chips precoated with polyethyleneimine (PEI) (Sigma-Aldrich) and laminin (Sigma-Aldrich).

### High density Microelectrode Array Electrophysiology (MEA)

MaxWell Biosystems high density microelectrode arrays (HD-MEAs) were used to assess electrophysiological activity of primary neuronal cultures. HD-MEA chips were incubated with a 1% Tergazyme solution (Alconox, White Plains, NY) for 2 h at room temperature then washed with DI H_2_O before being transferred to a beaker of 70% ethanol. The beaker containing the HD-MEA chips was transferred to the biosafety cabinet and allowed to incubate for 30 min at room temperature to sterilize the chips. HD-MEA chips were then washed with sterile water three times before pretreating with 1 ml of complete cell culture media consisting of Neurobasal supplemented with B27 (Gibco, ThermoFisher Scientific) and 10% horse serum (Gibco, ThermoFisher Scientific). The HD-MEA chips were allowed to incubate for two days in a humidified cell culture incubator at 37 C with 5% CO_2_. On the day of cell plating, the cell culture media was removed, and chips were washed three times with sterile water before being coated with 50 µl of polyethyleneimine (PEI, Sigma). Chips were placed back in the incubator for one hour, then the coating was aspirated, and the chips were washed three times with sterile water and allowed to dry in the biosafety cabinet for one hour. Laminin was then applied to the chips and placed back in the incubator until ready to plate cells ( ~ 1 h). The laminin coating was removed and 50 µl of primary neuronal cells isolated as previously described was immediately pipetted onto the HD-MEAs resulting in ~50,000 cells per chip. After incubating for 1 h in the cell culture incubator, an additional 600 µl of cell culture medium was added to each chip. The next day, 50% of the media in each chip was removed and replaced with cell culture maintenance media consisting of Dulbecco’s Modified Eagle Medium (DMEM) supplemented with GlutaMax, D-glucose and sodium pyruvate (Gibco, ThermoFisher Scientific) and 10% horse serum. Media changes happened every three days by removing 50% of the media and replacing it with an equal volume of cell culture media.

HD-MEA recordings were performed between day in vitro (DIV) 17 and DIV35, based on previously published literature [34–36]. The MaxWell Biosystems recording unit was sterilized with 70% ethanol, placed in the biosafety cabinet, and allowed to dry for 30 min. The recording unit was then transferred to an incubator at 37 °C with 5% CO_2_ for at least two hours prior to recording to allow for temperature equilibration. Performing MEA recordings inside the incubator ensured consistent temperature and pH values of the cell culture media while performing various scans. To assess neuronal electrical activity across the entire electrode array, the “Activity Scan Assay” module in the MaxLab Live software (MaxWell Biosystems AG, Zurich, Switzerland) was used. The “Full Scan” electrode configuration was used to measure neuronal activity from all 26,400 electrodes for 30 s each. After the Activity Scan was complete, the “Network Assay” module was used to assess network activity or axonal features. Network electrical activity was recorded by selecting a subset of 1024 electrodes with the highest firing rate from the corresponding chip’s Activity Scan and measured simultaneously for 300 s. Custom code was written to analyze the data using MatLab R2021.

### Experimental design and statistical analysis

Data were analyzed with GraphPad Prism. Group sizes were chosen based on experience and power analyses, using Cohen’s d (Silverman and Crawley, 2014; Silverman et al., 2015; Sukoff Rizzo and Silverman, 2016). Statistical testing was performed using established assay-specific methods, including Student’s *t*-test for single parameter comparisons between genotypes, and one-way or two-way repeated-measures ANOVA for comparisons across time points. All significance levels were set at *p* < 0.05 and all t-tests were two-tailed. Group sizes were chosen based on experience and power analyses [17, 24, 37]. Significant ANOVAs were followed by Bonferroni-Dunn or Holm-Sidak posthoc testing. Outliers were identified using the Grubb’s test for outliers. Behavioral analysis passed distribution normality tests, was collected using continuous variables and thus was analyzed via parametric analysis in all assays. For all behavioral analyses, variances were similar between groups and data points within two standard deviations of the mean were included in analysis. Finally, sex differences were not observed in any behavioral assay previously and thus sexes were combined in the main text, while Figures S2-S5, in the supplementary material illustrate both sexes, and a lack of sex differences.

## Results

### Reduced SynGAP1 protein levels in *Syngap1*^+/−^ mutant mice

**Fig. 1:**
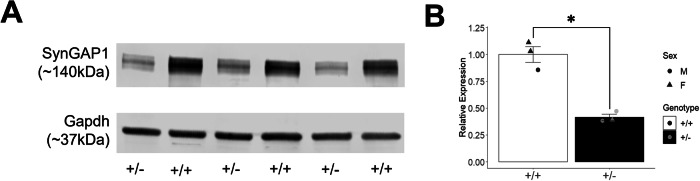
Syngap1^+/−^ mice had a significant decrease in the protein expression of SynGAP1 compared to Syngap1^+/+^ mice. **A** SynGAP1 and Gapdh protein expression in *Syngap1*^*+/+*^ and *Syngap1*^*+/−*^ mice at PND42. Western Blot analysis of SynGAP1 (140 kDa) shows a decrease in expression of SynGAP1 in the *Syngap1*^*+/−*^ mice. **B** Quantification of SynGAP1 protein expression normalized using Gapdh constitutive expression. SynGAP1 protein expression was significantly decreased to 41% expression in Syngap1^+/*−*^ brains, compared to 100% expression in Syngap1^+/+^ littermate controls. *Syngap1*^*+/−*^ brains as compared to *Syngap1*^*+/+*^ littermates. Data was analyzed using a Student’s t-test and is expressed as mean ± S.E.M. *P = 0.0051. ( *Syngap1*^+/+^ *N* = 3, *Syngap1* *N* = 3).

Western blot analysis was performed on PND42 cortex lysates from *Syngap1*^+/−^ and wildtype (WT) age and sex matched littermate controls, using a previously validated antibody to measure the level of total SynGAP1 protein expression relative to GAPDH [12, 14]. Western blot identified two bands matching previously described SynGAP1 isoforms, and we quantified the predominant band that corresponds to larger isoforms (Fig. 1A, *N* = 3, full gel image in Fig. S1). The heterozygous *Syngap1*^+/−^ mutants SynGAP1 protein reduced to 41% of normalized wildtype expression (Fig. 1B; *t*_(3)_ = 7.3937, *P* = 0.0051).

### Increased locomotion and impaired cognition in *Syngap1*^+/−^ mice

**Fig. 2:**
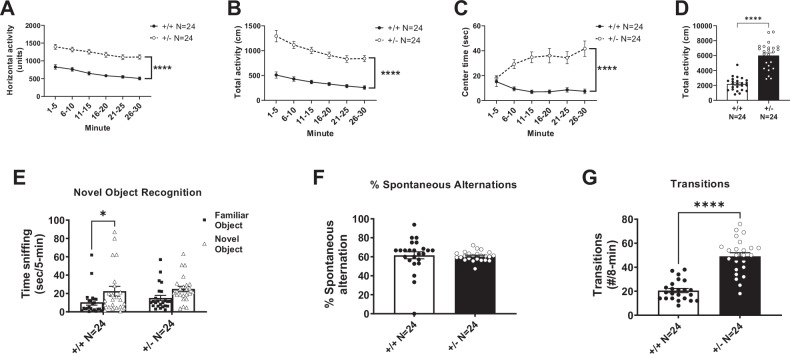
Syngap1^+/−^ mice showed elevated motor activity and impaired cognition when behavior was assessed. **A** Horizontal activity, (**B**) total activity, and (**C**) center time were all significantly increased when compared to *Syngap1*^*+/+*^ mice. Horizontal activity, total activity, and center time were analyzed using a repeated measures ANOVA. **D** Total activity over 30 min was compared with a Student’s t-test. **E** *Syngap1*^*+/−*^ mice showed no preference for the novel object when compared to wildtype mice. **F** In the spontaneous alternation task, there was no difference in the percentage of alternations between wildtype and *Syngap1*^*+/−*^ animals. **G** *Syngap1*^*+/−*^ mice made significantly more total transitions between arms in the spontaneous alternation task when compared to wildtype animals. Groups compared with a Student’s t-test. Data are expressed as mean ± S.E.M. * = *P* < 0.05, **** = *P* < 0.0001.

*Syngap1*^+/−^ mice displayed hyperactivity in the open field assay. Significantly more locomotion was observed in *Syngap1*^+/−^ mice when compared to *Syngap1*^+/+^ mice at every time bin of a 30 min session by horizontal activity (Fig. 2A; *F*_(1, 46)_ = 87.18, *P* < 0.0001 (main effect); 1–5 min, *P* < 0.0001; 6–10 min, *P* < 0.0001; 11–15 min, *P* < 0.0001; 16–20 min, *P* < 0.0001; 21–25 min, *P* < 0.0001; 26–30 min, *P* < 0.0001). Similar elevated levels of activity were observed at every time point when total activity was assessed (Fig. 2B; *F*_(1, 46)_ = 88.90, *P* < 0.0001 (main effect); 1–5 min, *P* < 0.0001; 6–10 min, *P* < 0.0001; 11–15 min, *P* < 0.0001; 16–20 min, *P* < 0.0001; 21–25 min, *P* < 0.0001; 26–30 min, *P* < 0.0001). Center time was significantly increased in *Syngap1*^+/−^ mice when compared to *Syngap1*^+/+^ mice during five of the six five-minute time bins (Fig. 2C; *F*_(5, 230)_ = 8.573; *P* < 0.0001 (main effect), 6–10 min, *P* = 0.0001; 11–15 min, *P* < 0.0001; 16–20 min, *P* = 0.0003; 21–25 min, *P* = 0.0002; 26–30 min, *P* < 0.0001). Taken together with the increased time spent in the open arms and transitions in the elevated plus maze (Fig. S2), the elevated center time suggests a reduced anxiety-like phenotype, as well as a hyperactivity phenotype. When summed over the 30 min session, the total activity of the *Syngap1*^+/−^ was significantly higher than the *Syngap1*^+/+^ mice (Fig. 2D; *t*_(46)_ = 9.429, *P* < 0.0001).

Cognitive abilities were tested in both novel object recognition task (long-term memory) and the Y-maze (working learning and memory). We observed a reduced preference for the novel object in the *Syngap1*^+/−^ mice, when compared to investigation of the familiarized object, indicating a cognitive deficit (Fig. 2E; *t*_(43)_ = 1.572, *P* = 0.1234). As expected, the *Syngap1*^+/+^ mice spent significantly more time investigating the novel object compared to the familiar object (Fig. 2E; *t*_(43)_ = 2.512, *P* = 0.0158). When transformed, as commonly done with drug treatment evaluations, the novel object did not reach significance using the index (data not shown, *t*_(44)_ = 0.1618, *P* > 0.05). In the Y-maze, *Syngap1*^+/−^ mice did not significantly differ from WT mice in the percentage of triads (Fig. 2F; *t*_(46)_ = 0.2335, *P* = 0.8164). However, the *Syngap1*^+/−^ mice made significantly more transitions between arms in the Y-maze when compared to *Syngap1*^+/+^ mice, providing further evidence of their hyperactivity phenotype (Fig. 2G; *t*_(45)_ = 8.154, *P* < 0.0001).

### Altered in vivo electroencephalography in *Syngap1*^**+/−**^ mice

**Fig. 3:**
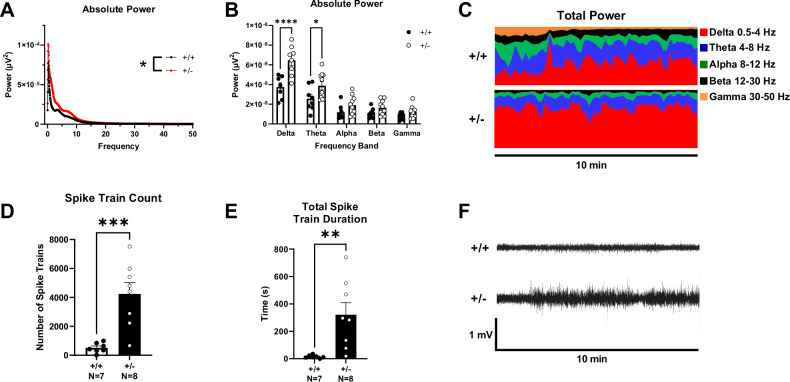
Syngap1^+/−^ mice displayed elevated delta and theta power. Surface EEG was collected using wireless telemetry and power spectral densities were compared at delta (0.5–4 Hz), theta (4–8 Hz), alpha (8–12 Hz), beta (12–30 Hz), and gamma (30–50 Hz) frequencies. **A** Power spectral density collected from *Syngap1*^*+/−*^ mice was increased compared to *Syngap1*^*+/+*^ mice. **B** Delta and theta frequency power were significantly increased in *Syngap1*^*+/−*^ mice. **C** Representative power distribution of *Syngap1*^*+/+*^ mice and *Syngap1*^*+/−*^ mice over a 10 min period illustrates elevated delta and theta power in *Syngap1*^*+/−*^ mice. **D** *Syngap1*^*+/−*^ mice exhibited elevated spike train counts. **E** *Syngap1*^*+/−*^ mice displayed significantly increased total spike train duration. **F** Representative EEG traces of *Syngap1*^*+/+*^ and *Syngap1*^*+/−*^ mice recorded over 10 min. Data were analyzed using a two-way ANOVA between genotype and frequency and Sidak’s post hoc analysis where applicable (**A**, **B**), or with Student’s t-test (**D**, **E**). Data are expressed as mean ± S.E.M. * = *P* < 0.05, ** = *P* < 0.01, *** = *P* < 0.001 **** = *P* < 0.0001.

A wireless telemeter system was used to measure electroencephalographic activity. *Syngap1*^+/−^ mice displayed an elevated absolute power spectral density (PSD) compared to *Syngap1*^+/+^ mice when measured for 72 h and compared with a two-way ANOVA between genotype and frequency (Fig. 3A; *F*_(1, 13104)_ = 134.5, *P* < 0.0001). When observing differences in different power bands, *Syngap1*^+/−^ mice displayed elevated Delta power (0.5–4 HZ) and Theta power (5-9 HZ) when compared with a two-way ANOVA between genotypes and subsequent post hoc analysis (Fig. 3B; *F*_(1, 16)_ = 8.598, *P* = 0.0098; *P* < 0.0001 (Delta), *P* = 0.0287 (Theta)). Delta power and theta power bands were elevated in *Syngap1*^+/−^ mice in representative total power distributions (Fig. 3C). We observed a significantly increased spike train count in *Syngap1*^+/−^ mice compared to *Syngap1*^+/+^ mice (Fig. 3D; *t*_(13)_ = 4.396, *P* = 0.0007). The duration of spike trains was also significantly elevated in *Syngap1*^+/−^ mice compared to *Syngap1*^+/+^ mice (Fig. 3E; *t*_(13)_ = 3.215, *P* = 0.0068). *Syngap1*^+/−^ mice displayed higher signal than *Syngap1*^+/+^ mice when viewing raw EEG output (Fig. 3F).

**Fig. 4:**
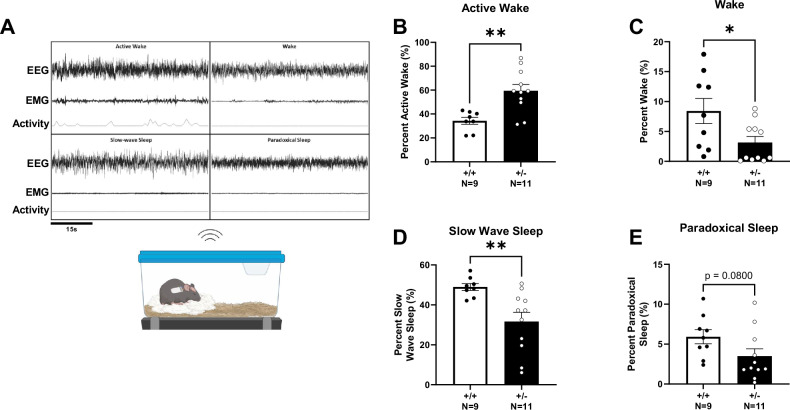
Syngap1^+/−^ mice displayed altered EEG sleep signatures. **A** Schematic of wireless telemetric recording system and representative signals from the four sleep stages. EEG signal is recorded while mice are in their home cage and allowed to move freely. **B** Active wake percentage determined from EMG recording and integrated accelerometer when subject displayed movement while awake. *Syngap1*^*+/−*^ mice displayed elevated active wake time. **C** Percentage of wake across 72 h where animals were not actively moving. *Syngap1*^*+/−*^ mice showed reduced wake compared to *Syngap1*^*+/+*^ mice. **D** Slow wave sleep percentage was decreased in *Syngap1*^*+/−*^ mice. **E** Paradoxical sleep percentage from 72 h of recording trended lower in *Syngap1*^*+/−*^ mice. Data were analyzed using Student’s unpaired t-test. Data are expressed as mean ± S.E.M. * = *P* < 0.05, ** = *P* < 0.01.

We characterized four sleep stages and found alterations in *Syngap1*^+/−^ mice (Fig. 4A). We first assessed active wake, during which the subjects are awake and moving measured by EMG signal and activity. *Syngap1*^+/−^ mice displayed an increased percentage of time in the active wake stage (Fig. 4B; *t*_(17)_ = 3.659, *P* = 0.0019). We then assessed the percent of time in the wake stage where the subjects are awake but not active. *Syngap1*^+/−^ mice had a significantly reduced percentage of wake time compared to *Syngap1*^+/+^ controls (Fig. 4C; *t*_(18)_ = 2.398, *P* = 0.0275). Next, we examined sleep characteristics and found a significant reduction in the percent of slow wave sleep in *Syngap1*^*+/−*^ mice compared to *Syngap1*^*+/+*^ mice (Fig. 4D; *t*_(17)_ = 3.060, *P* = 0.0071). Although paradoxical sleep was not significantly affected, *Syngap1*^*+/−*^ mice trended toward a lower percentage of time in paradoxical sleep (Fig. 4E; *t*_(18)_ = 1.856, *P* = 0.0800).

### Elevated electrophysiological activity in cultured primary neurons from *Syngap1*^+/−^ mice

**Fig. 5:**
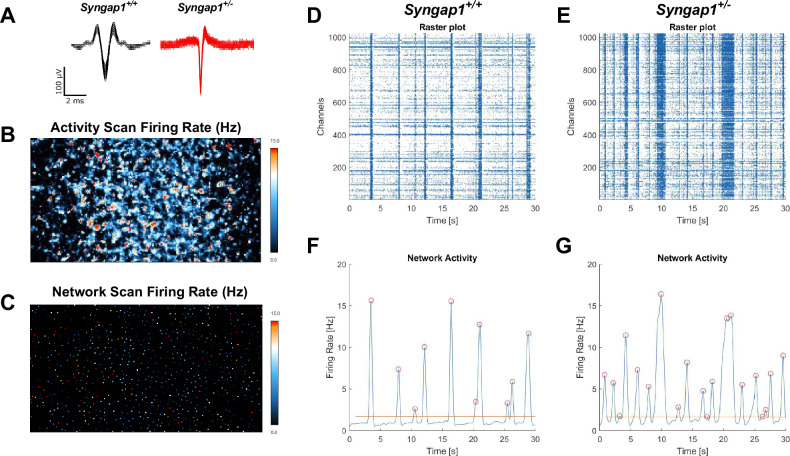
Primary neurons from Syngap1^+/−^ mice displayed increased network firing activity when measured with high-density microelectrode arrays (HD-MEAs). **A** Overlaid raw action potential traces. **B** Firing rate from the entire chip area. **C** 1024 electrodes with the highest firing rates were chosen to conduct a “Network Activity Scan”. **D** Representative raster plot and **F** network activity plot of *Syngap1*^*+/+*^ primary neurons. **E** *Syngap1*^*+/−*^ raster plot and (**G**) network activity plot exhibit increased activity. 1024 electrodes were recorded simultaneously and plotted on the y-axis of raster plots.

**Fig. 6:**
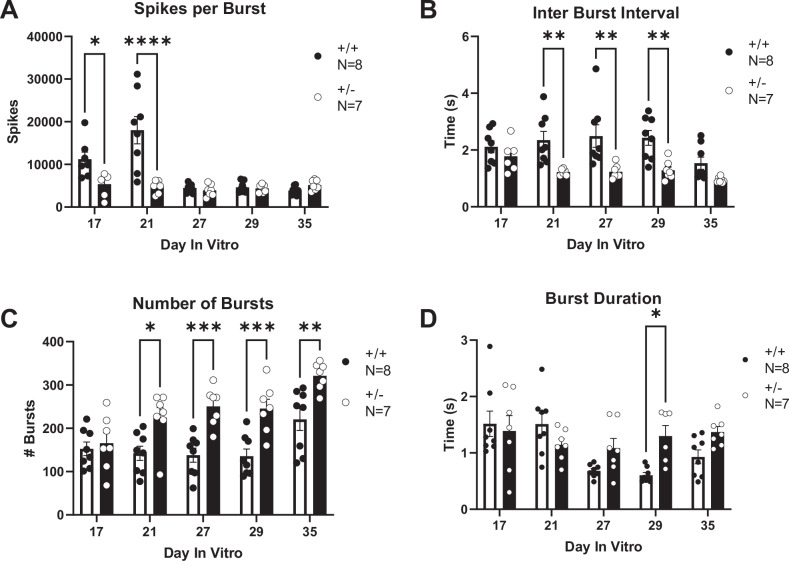
Syngap1^+/−^ mice displayed increased bursting activity when measured on HD-MEAs. **A** Spikes per burst in *Syngap1*^*+/+*^ and *Syngap1*^*+/−*^ mice. **B** Inter burst interval (IBI) was measured as time between burst peaks. A reduced IBI was observed in *Syngap1*^*+/−*^ animals on DIVs 21, 27, and 29. **C** The total number of bursts over the five-minute recording was compared. *Syngap1*^*+/−*^ mice exhibited an increased number of bursts on all days after DIV21. **D** *Syngap1*^*+/−*^ mice had a longer burst duration on DIV29 when compared to *Syngap1*^*+/+*^ mice. Data were analyzed using a two-way ANOVA. Data are expressed as mean ± S.E.M. * = *P* < 0.05, ** = *P* < 0.01, *** = *P* < 0.001 **** = *P* < 0.0001.

Electrophysiological activity was assessed in cultured primary neurons from both *Syngap1*^*+/−*^ and *Syngap1*^*+/+*^ mice on high-density microelectrode arrays. Overlaid raw signal traces show different action potential shapes between neurons from *Syngap1*^*+/−*^ and *Syngap1*^*+/+*^ mice (Fig. 5A). Firing rate from the entire chip area was assessed following an “Activity Scan” of all 26,400 electrodes for 30 s (Fig. 5B). Electrodes with the highest firing rate were then chosen to perform a “Network Activity Scan” where the activity from 1024 electrodes was recorded simultaneously for five minutes (Fig. 5C). Raster plots were generated to visualize bursting events from the simultaneously recorded electrodes. Vertical lines visible on the raster plots indicate coordinated, synchronized bursting activity and were visually elevated in *Syngap1*^*+/−*^ mice (Fig. 5E) compared to *Syngap1*^*+/+*^ mice (Fig. 5D). Activity was plotted following Gaussian convolution of the spiking data to quantify bursting events for *Syngap1*^*+/+*^ mice (Fig. 5F) and *Syngap1*^*+/−*^ mice (Fig. 5G). *Syngap1*^*+/−*^ mice emitted fewer spikes per burst when compared to *Syngap1*^*+/+*^ mice (Fig. 6A; *F*_(1, 13)_ = 11.56, *P* = 0.0047), on DIV17 (*P* = 0.0418) and DIV21 (*P* < 0.0001). Inter-burst interval was significantly reduced in the *Syngap1*^+/−^ mice (Fig. 6B; *F*_(1, 76)_ = 20.33, *P* = 0.0024) on DIV21, DIV27, and DIV29 when analyzed with Sidak’s multiple comparison test following two-way ANOVA (DIV21, *P* = 0.0068; DIV27, *P* = 0.0010; DIV29, *P* = 0.0035). The reduced inter-burst interval in the *Syngap1*^*+/−*^ mice aligns with the increased number of bursts (Fig. 6C; *F*_(1, 78)_ = 32.86, *P* < 0.0001) observed on DIV21, DIV27, DIV29, and DIV35 when compared to neurons from *Syngap1*^*+/+*^ mice following post hoc analysis (DIV21, *P* = 0.0129; DIV27, *P* = 0.0003; DIV29, *P* = 0.0004; DIV35 *P* = 0.0013). We also measured an increased burst duration on DIV29 in *Syngap1*^*+/−*^ neurons compared to *Syngap1*^*+/+*^ neurons (Fig. 6D; *F*_(1, 64)_ = 4.166, *P* = 0.0454; DIV29, *P* = 0.0229). Together, data indicate that cortical neurons from *Syngap1*^*+/−*^ exhibit burst firing in greater number and with shorter time between bursts than cortical neurons from *Syngap1*^*+/+*^ mice at comparable time points.

## Discussion

A myriad of NDDs result from the loss of proteins of the postsynaptic density (PSD), including Shanks [38, 39], Homers [40–42], mGluRs [43], and SynGAP1. SynGAP1 is the major neuronal specific RasGAP that binds to the PSD-95, a major scaffolding protein of the PSD, localized to excitatory synapses, and primarily expressed in the brain [1, 2]. Thus it is not surprising that perturbations in SynGAP1 expression result in a pathological NDD [2, 3, 44]. We observed robust behavioral hyperactivity in the open field arena, impairments in novel object recognition, and reduced anxiety-like behavior in *Syngap1*^*+/−*^ mice compared to *Syngap1*^*+/+*^ littermate controls, using an F1 hybrid, as the background strain. Our study extends earlier results on sleep, with greater precision, and over an extended period by utilizing wireless, untethered EEG acquisition and 72 h. Analysis to identify alterations in sleep architecture by reductions in slow wave sleep, and wake and increased active wake [27, 45]. Additionally, this is the first report of high-density microelectrode analysis (HD-MEA) in any model system of SRID, illustrating differing burst patterns in neurons with reduced *Syngap1*, compared to WT neurons from age and sex matched brains. Our study is novel notwithstanding the lack of a new animal model being created. Alternatively, we combined newer technologies and performed previously unexecuted analysis, in vitro and in vivo, in parallel, in an existing model, to “bridge critical gaps in translation.”

Advantages of utilizing an F1 background strain, include hybrid vigor, which eliminates the classic congenic C57BL/6J background strain’s resistance to typical seizure induction methods [29, 46, 47]. A comprehensive behavioral battery performed at the neurobehavioral phenotyping laboratory at the Jackson Laboratory found the 129S1-C57BL/6 J F1 hybrid to be behaviorally indistinguishable from C57BL/6 J. Moreover, identical observations, to those herein, were reported in *Shank1* mutant mice, where lethality was observed when the mutation was on a congenic C57BL/6 J background, yet excellent propagation, and indistinguishable behavioral scores between *Shank1*^+/−^ and WT, when assayed on the BL/6 J and 129S1Ev F1 hybrid [26, 39, 48]. In our case, investigating the construct valid *Syngap1* mutant mice on C57BL/6 J or 129S1 background strains alone, would have prevented data with ample power for statistical conclusions and/or reduced the clarity of our conclusions. Surprisingly, Nakajima et al. [27] was able to perform an exhaustive, powered analysis using the Huganir mouse backcrossed for 10 generations by Dr. Seth Grant’s laboratory with C57BL/6 J. Unconventionally, their behavioral analysis began at 53 weeks and ended at 92 weeks or 2 years of age. This was unusual for behavioral studies of mouse models of NDDs, since mice live, in the wild, ~3–4 months, while mice in the laboratory live ~18–24 months. Aging and neurodegenerative work usually begins at 9 months of age, with 2 years utilized as a typical final timepoint, chosen for attrition rates [25, 49, 50]. Females cease reproductivity at ~6 months of age and thus the Nakajima behavioral study utilized extremely aged mice. This is also evident by points in their data, such as the very short time for the WT to fall off the rotarod ~40 s whereas during the 300 s test, the average ranges from ~120 to 200 s, standard for adults.

Sleep disturbances are a significant translational phenotype in synaptopathies, such as Phelan McDermid Syndrome (mutation in or loss of *Shank3*) and SRID [51]. In a 3-year-old child and the *Syngap1* mutant mice [29, 52], similar progressive changes in the sleep architecture over 24 a hr. period were reported. Using a 24 h assessment of nocturnal rhythms, WT mice at PND60 and PND120 had less “awake” time than *Syngap1*^*+/−*^ mice at similar ages [45]. Our report extends this result and illustrates a generalization of sleep alterations across mouse background strain genetics. We utilized four sleep stages via EEG wave frequency assessment, EMG and an accelerometer [45]. A one-of-a-kind *SYNGAP1* rat model exists, published under the *Syngap*^*+/Δ−GAP*^ nomenclature, as the calcium/lipid binding domains and GTPase-activating protein domains were deleted, making this rat a rasopathy model, and it’s synaptopathic relevance is unknown, to date. *Syngap1* encodes multiple isoforms that are essential for neuronal and brain development, signaling and survival. All isoforms share a central 5’ region comprised of a calcium/lipid binding domain (C2) and a GTPase activating protein (GAP) domain that function together to regulate the intrinsic GTPase activity of the small G proteins Ras and Rap [53–56]. Some isoforms function as a scaffolding protein, anchoring AMPA receptors to the PSD through the regulation of transmembrane AMPA receptor-associated proteins, but these isoforms are intact in the rat model, *Syngap1*^+/Δ-GAP^, yet dysfunctional in the *Syngap1*^*+/−*^ mice presented, in this report, which is supported by our in vitro HD-MEA findings. In the rat study, sleep was analyzed via multi-electrode EEG recordings using an EEG probe placed on the skull with reference aligned over Bregma using 6 h recording periods, 3 h. after “lights on” using ‘zeitgeber time’ (ZT) [57]. Scoring criteria for visual classification were based on accelerometer and EEG characteristics, like the methodology herein, however, without the “active wake” distinction, and using the NREM nomenclature for wake that is not “active” and slow-wave sleep. Our automated sleep module integrated the three outcomes of EEG, EMG, and accelerometer, while the rat model used 2 of the 3 inputs. Additionally, the former work used group sizes of 4 per genotype and did not delineate the sex of the subjects, whereas our Cohen’s d power analysis required 6–8 subjects. Using the earlier described methodology, it was reported that *Syngap*^+/Δ−GAP^ rats spent an equivalent percentage of time in all states when compared with WT littermate controls [57]. Our report found reduced amount of time in slow wave sleep and wake, and identified substantially elevated delta power (*p* < 0.0001) and significantly elevated theta spectral power (*p* < 0.05). Direct comparison of our data presented herein to the sleep data from the rat model is complicated given the vastly different methodologies, species, EEG acquisition time, channels utilized to acquire data, and analysis of the signal. The consensus of these studies is that sleep is a powerful translational predictor for a future clinical trial for SRID, as has been demonstrated in other rare genetic NDDs [58–62].

EEG recordings in NDDs show potential for identifying multiple, clinically translatable, objective biomarkers, with the power to be diagnostic biomarkers, as well as biomarkers that track progress of novel therapeutic strategies. When spontaneous recurring seizures were observed by visual scoring of a 24 h video EEG, few seizures were observed in the *Syngap1*^*+/−*^ mice until PND120 (4 months of age), an age at which EEG seizures greatly increased [45]. Our data corroborate work using the indices of spiking and spike trains, analyzed using the same methods as other laboratories (i.e., Baylor College of Medicine) [63, 64]. We also utilized the oscillatory power to obtain power spectral densities (PSD) of each frequency wave, identifying greater absolute power in *Syngap1*^*+/−*^ mice, and elevated delta and theta power, compared WT age and sex matched control subjects. Elevated delta power is currently being used as a biomarker in clinical trials for other genetic NDDs, such as the Angelman Syndrome trial using GTX-102 [31, 64–68]. Increased spike trains in vivo during EEG show similar activity to the HD-MEA outputs of increased bursts and shorter latency between bursts with *Syngap1*^*+/−*^ primary neurons. This is the first report of HD-MEAs in a model system for SRID, illustrating with clarity that *Syngap1*^*+/−*^ neurons exhibit differing burst patterns, compared to WT neurons from age and sex matched control brains, identifying a functional physiology outcome that bridges our in vitro studies to our in vivo results. Although we observed differences in the number of spikes per burst at earlier DIVs, changes to the maturing neuronal network might have caused this trend to disappear at later time points. When observing burst duration, *Syngap1*^*+/−*^ neurons showed a statistically significant difference at DIV 29, however the same effect was trending toward significant at DIVs 27 and 35, similar to the effects seen with inter-burst interval and number of bursts. Critical to translation, this technology can be used to record from neural stem cells generated from human iPSCs, which may serve to further bridge the translational gap of mouse to human [35]. Here, we utilized HD-MEA containing 26,400 electrodes that has ability to simultaneously record 1024 discrete electrodes for label-free, comprehensive, electrophysiological neuronal cell recording of over 3–4 weeks in culture [34, 35].

As a negative regulator of excitatory neurotransmission, overexpression of *Syngap1* results in a dramatic loss of synaptic efficacy as well as enhanced synaptic transmission following SynGAP1 disruption by RNA interference [4]. This work is vital, proving that SynGAP1 levels are modifiable and SynGAP1-deficient synapses do not lack the potential to be adjusted. Added support for the theory that SynGAP1 is tunable comes from recent work that illustrates that SynGAP1 is a downstream target of MAPK interacting protein kinases 1 and 2 (Mnk1/2) [69], which regulate a plethora of functions, presumably via phosphorylation of substrates, including eukaryotic translation initiation factor 4E (eIF4E). Reducing the level of SynGAP1 reversed behavioral learning and memory deficits in a Mnk 1/2 double knockout mouse model, leading to the theoretical proposition that the Mnk–SynGAP1 axis regulates memory formation and functional outcomes [69].

Key questions that remain for all NDDs include age of restoration, and “critical windows”. Many have described that rescues are possible as adults in Fragile X [70, 71], Rett [72, 73], Phelan-McDermid [74], and Angelman Syndromes [20, 22, 75]. However, others have argued that intervention must occur early in life for reversal or rescue of behavioral phenotypes, to be possible in genetic NDDs [76]. In fact, prenatal intervention theories are now being explored [77]. Earlier work with *Syngap1* illustrated hardwiring of neural circuitry manifesting as lifelong impairments [78, 79]. Nonetheless, crucial to the data herein, is that re-expression of *Syngap1* by genetic reversal exhibits a complete alleviation of electrophysiological and cognitive behavioral phenotypes in a genetic inducible mouse model [10]. Recovery required nearly full expression of a second allele of *Syngap1* for alleviation of phenotypes. Thus, exploration regarding potency, target engagement, and PK/PD will be required for regulatory meetings and translatability. Our laboratory is currently assessing nuanced SynGAP1 alterations using an ELISA assay over semi-quantitative Western blots or RNA levels only, which can lack predictability, as RNA is not always translated 1:1 to protein. Genetic reversal was illustrated when re-expression was localized to glutamatergic neurons, which contribute significantly but not in isolation to the phenotypes reported herein. It is currently not known if other neuronal subtypes are also sufficient to drive the reported abnormalities in these mice, however, our development of targeted therapeutics, under current investigation in this construct valid model are addressing that exact question.

In summary, over 20 years since its discovery, we have built on earlier work to identify a combinatorial and corroborative report on electro- and neurophysiological biomarkers for SRID precision therapeutic development [57, 78, 10, 80, 81]. Our key novelty, from prior reports, lies in the reporting and analytic combination of: i) in vitro HD-MEA recording and analysis using dissociated and plated cortical neurons from WT and *Syngap1*^+/−^ cortices, in parallel, with ii) in vivo wireless, cortical EEG, capturing cortical circuitry and bridging 2D in vitro electrophysiological properties with 3D, live, awake, behaving, in vivo neurophysiological outcomes. To date, 2D SRID cellular modeling work had been limited to traditional patch clamp electrophysiology in the various mouse and rat models [57, 81]. This is the first report using HD-MEAs in neurons lacking *Syngap1*/SynGAP1 and highlighting increased network activity by a greater number of bursts with less time between bursts, compared to neurons from WT cortices. Second, this is the first report of EEG in awake, behaving mice, in their home cage, collected with wireless telemetry over several light/dark cycles. Translational EEG analysis has not been performed in any SRID rodent model, to date. Herein, we report elevated spiking, spike trains and disrupted sleep stages in mice lacking *Syngap1*, compared to WT age and sex matched controls. Finally, comprehensive behavior analysis reproduced earlier findings of hyperactivity and poor Y-maze performance, a key component of rigor in translational neuroscience, in addition to, identifying an extended number of behavioral assays on which mice lacking *Syngap1* performed more poorly, compared to WT age and sex matched controls. Our unique combination of in vitro and in vivo technologies has discovered cellular and functional phenotypes and neurophysiological biomarkers, with potential for advancing targeted therapeutics.

## Supplementary information

Supplemental Methods, Supplemental Results and Figures
