# Supplementary material for: Hyperexcitability and translational phenotypes in a preclinical mouse model of SYNGAP1-related intellectual disability
Source: Transl Psychiatry. 2024 Oct 2;14:405. doi: 10.1038/s41398-024-03077-6 (PMC11447000; doi:10.1038/s41398-024-03077-6)
Supplement: Supplementary file 1 — Supplemental Methods, Supplemental Results and Figures [file 41398_2024_3077_MOESM1_ESM.pdf]

Supplementary Results and Supplemental Figure Legend

Hyperactivity and reduced anxiety like phenotype

Anxiety -like activity was assessed using the elevated plus maze and the light-dark box tasks. An increased percentage of time spent in the open arms of the elevated plus maze was observed in the *Syngap1*$^{+/-}$ mice when compared to *Syngap1*$^{+/+}$ mice using a student's unpaired t-test (**Fig. S1A**; $t(46) = 2.471$, $P = 0.0172$) indicating reduced anxiety-like behavior. *Syngap1*$^{+/-}$ mice also showed heightened open arm entries (**Fig. S1B**; $t(46) = 4.104$, $P = 0.0002$) as well as total transitions between open and closed arms in the elevated plus maze when analyzed with an unpaired t-test (**Fig. S1C**; $t(46) = 5.240$, $P < 0.0001$). Similar elevated total entries were observed in the light-dark task with the *Syngap1*$^{+/-}$ mice showing a significantly increased number of total entries into the light space when compared to *Syngap1*$^{+/+}$ mice (**Fig. S1D**; $t(39) = 4.481$, $P < 0.0001$).

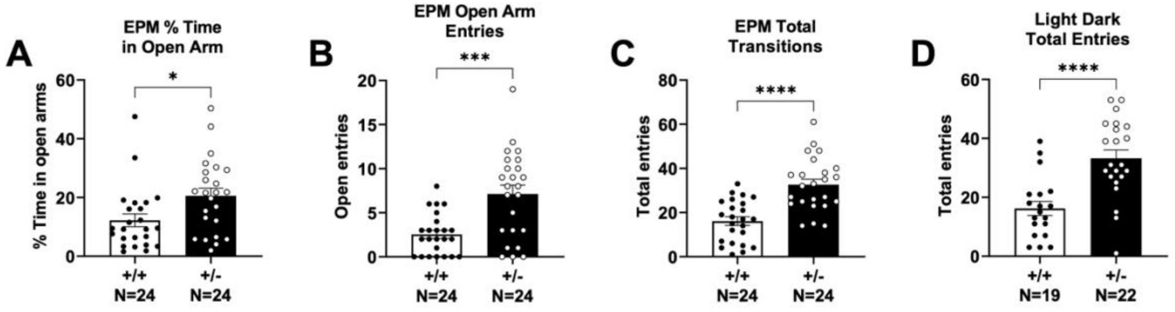

Figure S1. **Reduced anxiety-like behavior and hyperactivity when assessed in the elevated plus maze (EPM) and the light-dark conflict task. (A)** In the EPM, percent time spent on the open arms, **(B)** the total number of open arm entries, and the **(C)** total number of transitions were elevated in *Syngap1*$^{+/-}$ mice when compared to *Syngap1*$^{+/+}$ mice. **(D)** In the light-dark task, total transitions between chambers was elevated in the *Syngap1*$^{+/-}$ mice. Data are expressed as mean ± S.E.M. * = $P < 0.05$, *** = $P < 0.001$, **** = $P < 0.0001$ when analyzed with a student's unpaired t-test.

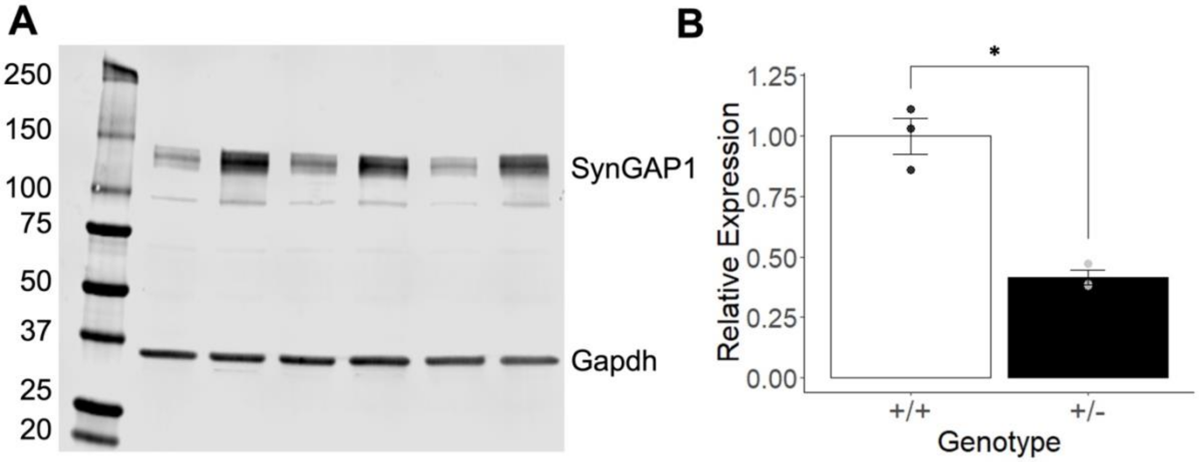

Figure S2. **Unedited western blot of SynGAP1 and Gapdh protein expression in *Syngap1*$^{+/+}$ (WT) and *Syngap1*$^{+/-}$ (HT) mice at PND42. (A)** A significant decrease in expression of Syngap1 was observed in the *Syngap1*$^{+/-}$ mice. Bands not at 140 kDa or 37 kDa are non-specific and do not show significant changes between genotypes. **(B)** Quantification of Syngap1 protein expression using Gapdh expression as normalization. Syngap1 protein expression was decreased to 41% of WT expression. Data was analyzed using a Student's t-test and is expressed as mean ± S.E.M. *P = 0.0051. (WT N = 3, HT N = 3)

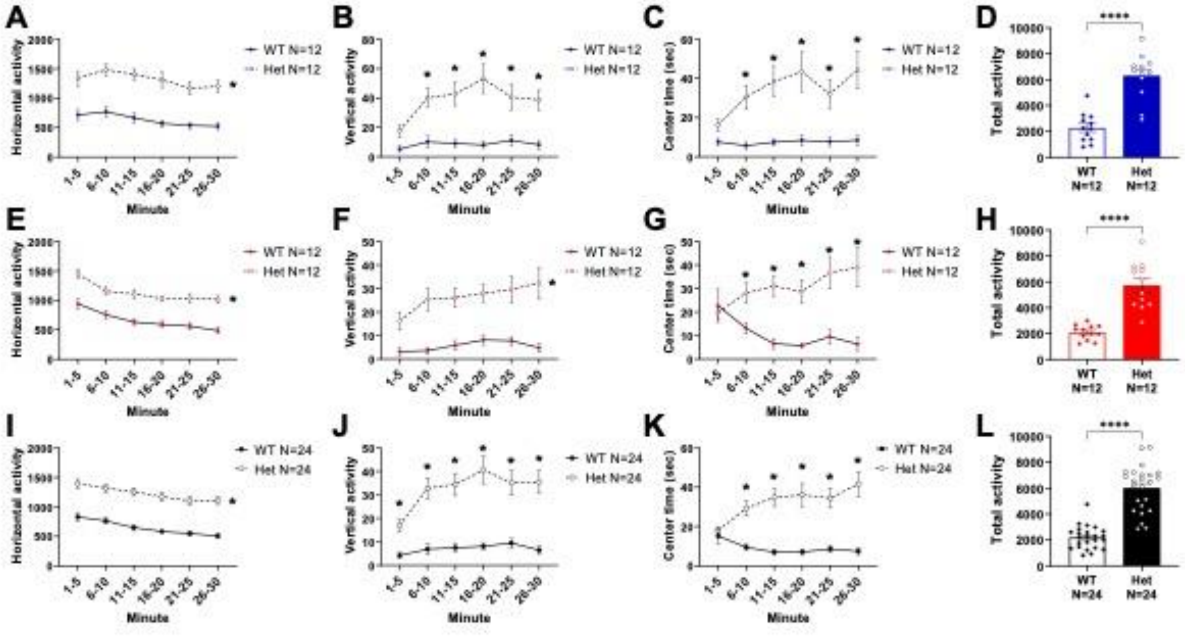

Figure S3. No observed gross motor differences in open field assay between *Syngap1*$^{+/+}$ (WT) and mice with a loss of Syngap1 expression, *Syngap1*$^{+/-}$ mice. (A,E,I) Time spent exploring the center of the open field apparatus over six 5-minute time bins for males (A, blue), females (E, red) and combined sexes (I, black). **(B,F,J)** Summed total activity for the 30-minute session for males (B, blue), females (F, red) and combined sexes (J, black). Data are expressed as mean ± S.E.M. * = $P < 0.05$, *** = $P < 0.001$, **** = $P < 0.0001$ when analyzed with a two-way ANOVA (center time) or with student's unpaired t-test (total activity).

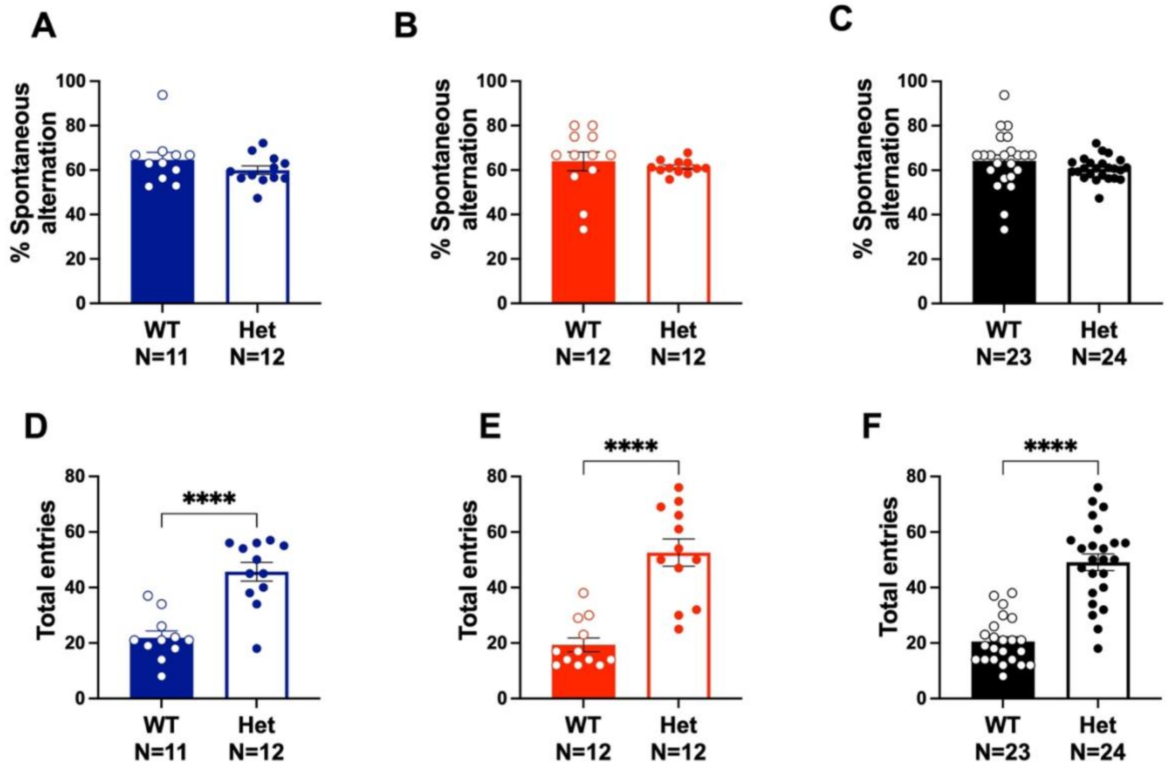

Figure S4. No observed sex differences in spontaneous alternation in the Y-Maze between *Syngap1*$^{+/+}$ (WT) and loss of Syngap1 expression *Syngap1*$^{+/-}$ mice. (A-C) Percent of spontaneous alternations in males (A, blue), females (B, red), and combined sexes (C, black). **(D-F)** Elevated arm entries in *Syngap1*$^{+/-}$ males (D, blue), females (E, red), and combined sexes (F, black) compared to *Syngap1*$^{+/+}$ mice. Data are expressed as mean ± S.E.M. * = $P < 0.05$, *** = $P < 0.001$, **** = $P < 0.0001$ when analyzed with student's unpaired t-test.

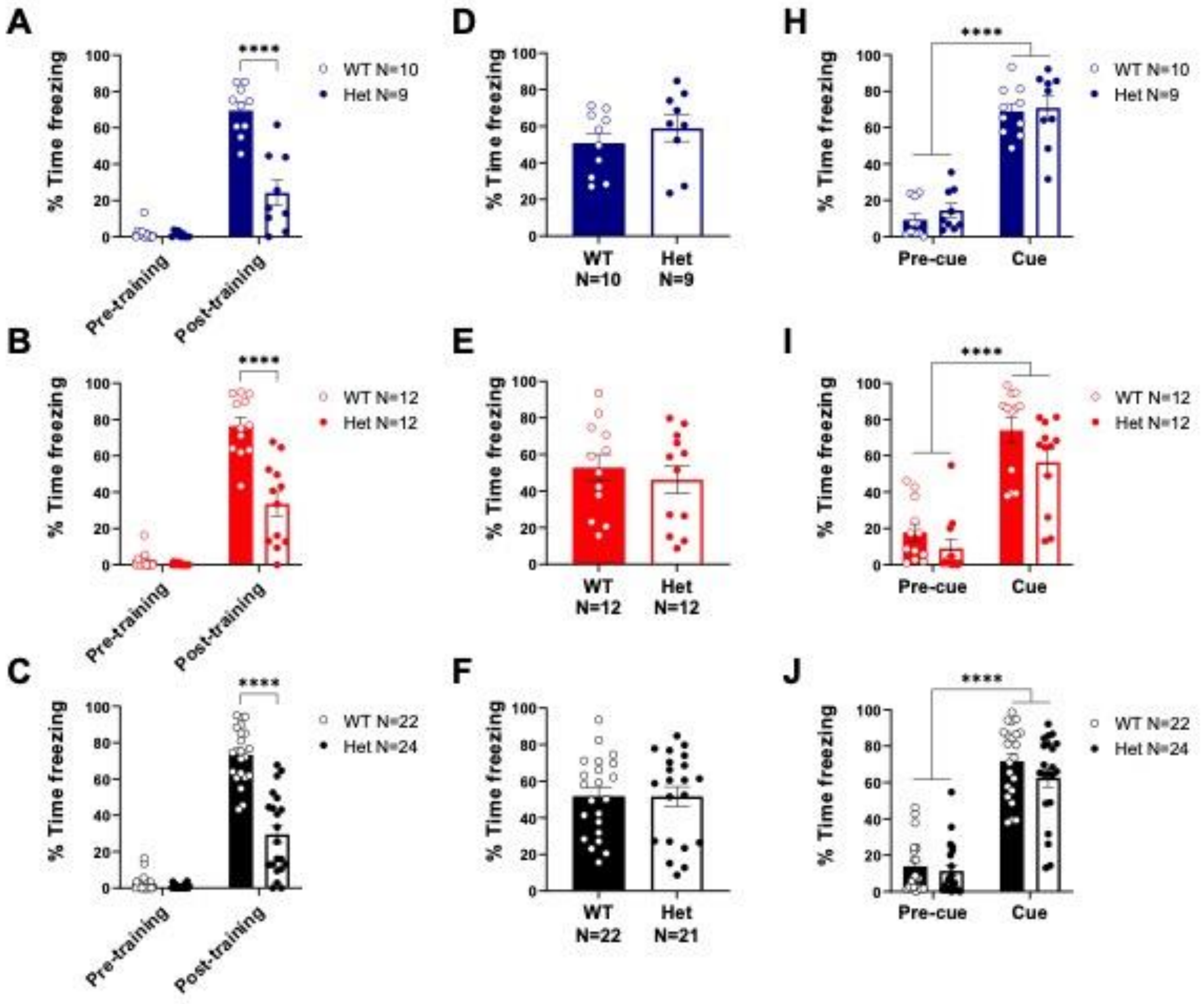

Figure S5. No observed sex differences in fear conditioning learning between *Syngap1*$^{+/+}$ (WT) and loss of Syngap1 expression, *Syngap1*$^{+/-}$ mice. (A-C) Percent of time spent freezing pre-training and post-training for males (A, blue), females (B, red), and combined sexes (C, black). **(D-F)** Total percent time freezing between *Syngap1*$^{+/+}$ (WT) and *Syngap1*$^{+/-}$ (HT) mice for males (D), females (E), and combined sexes (F). **(G-I)** Percent time freezing pre-cue and when cued for males (G), females (H), and combined sexes (I). Data are expressed as mean ± S.E.M. * = $P < 0.05$, *** = $P < 0.001$, **** = $P < 0.0001$ when analyzed with student's unpaired t-test.

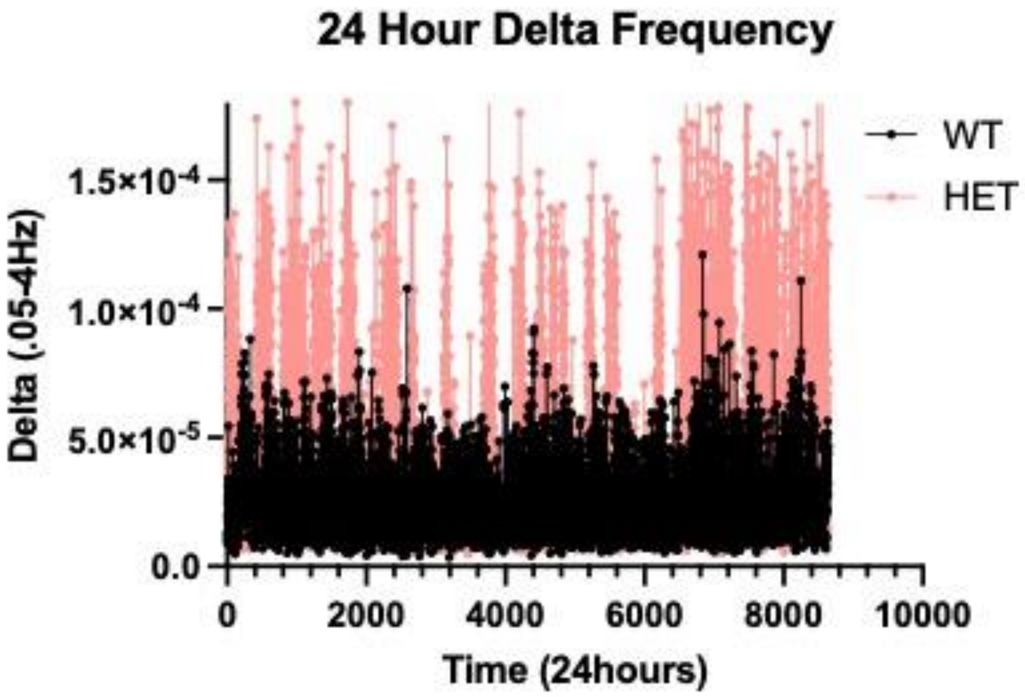

Figure S6. Representative tracing of delta spectral power across 24 hr. of the 72 hr. recording period in *Syngap1*$^{+/+}$ (WT) and loss of Syngap1 expression, *Syngap1*$^{+/-}$ mice.
